# How Childhood Maltreatment Contributes to Explaining Depressive Symptoms in Transgender and Gender-Diverse Individuals

**DOI:** 10.3390/healthcare14050558

**Published:** 2026-02-24

**Authors:** Arkadiusz Parker, Aleksandra M. Rogowska

**Affiliations:** Institute of Psychology, University of Opole, 45-052 Opole, Poland

**Keywords:** adverse childhood experience (ACE), cisgender, childhood trauma, depression, transgender identity, gender-diverse individuals

## Abstract

**Highlights:**

**What are the main findings?**

- Transgender and gender-diverse (TGD) individuals scored significantly higher in retrospectively assessed childhood trauma experiences and current depression symptoms than a sample of cisgender participants.
- Childhood trauma, especially emotional and sexual abuse, mediates the relationship between transgender and gender-diverse individuals and depression symptoms.

**What are the implications of the main findings?**

- When diagnosing depression in gender minority populations, it is crucial to consider childhood trauma.
- Intervention and preventive programs should be designed to improve the well-being of transgender people and those with diverse gender identities, and to support their families.

**Abstract:**

Background/Objectives: Transgender and gender-diverse (TGD) individuals experience disproportionately high rates of childhood trauma and depression; however, the mechanisms linking gender identity and depressive symptoms remain insufficiently understood. This study examines differences in depressive symptoms and childhood trauma between cisgender (CG) and TGD adults. It investigates whether specific subtypes of childhood maltreatment mediate the association between gender identity and depression. Methods: The cross-sectional online study included 249 participants aged 18–72 years (*M* = 30.85, *SD* = 12.72), including 144 CG (75 women and 69 men) and 105 TGD individuals (44 transgender and 61 gender diverse individuals). Depression symptoms were assessed using the nine-item Patient Health Questionnaire (PHQ-9), while childhood trauma experiences were measured using the Childhood Trauma Questionnaire–Short Form (CTQ-SF). Results: The independent-sample Student’s *t*-test showed that TGD participants reported significantly higher levels of depressive symptoms and all forms of childhood trauma than cisgender individuals. Mediation analyses indicated that overall childhood trauma partially mediated the association between gender identity and depression. In parallel mediation models, emotional abuse emerged as the primary statistical mediator, with sexual abuse showing a smaller indirect effect. Conclusions: The findings extend prior prevalence-focused research by identifying specific childhood trauma pathways associated with depressive symptoms in TGD adults. Experiencing traumatic events during childhood may be a key factor contributing to an increased risk of depression in adulthood, particularly among the TGD population. Therefore, intervention and prevention programs should target TGD individuals and their families to minimize the risk of adverse childhood experiences and mental health disorders. The results underscore the importance of trauma-informed and gender-affirming mental health care and highlight emotional abuse as a particularly salient correlate of depression in this population.

## 1. Introduction

### 1.1. Gender Identity

Transgender and gender-diverse (TGD) identity refers to people whose internal sense of gender does not align with the sex assigned to them at birth. This term serves as an umbrella category, including transgender women and men, as well as non-binary, genderqueer, agender individuals, gender nonconforming individuals, and people with a fluid gender identity (where one’s identity fluctuates between different genders), who exist outside the cis-normative binary system [1]. In many studies, participants identify with more than one category simultaneously (e.g., transmasculine and non-binary), highlighting the fluidity and multidimensionality of gender identity, as well as the lack of rigid boundaries between categories. Although not all non-binary people identify as transgender, these groups share a common trait: a gender identity different from the one assigned at birth. As a result, they are often collectively included in research as the gender minority population [2].

The definition of transgender has undergone a substantial evolution from a pathologizing medical construct to an affirming, inclusive perspective that emphasizes the diversity of gender identities and expressions, moving away from treating transgender as a disorder [1]. Gender identity is understood as the internal, subjective sense of belonging to a particular gender category: male, female, both, neither, or an identity outside the gender binary. In the case of transgender people, this identity does not automatically align with the sex assigned at birth, which is determined based on anatomical features. People whose gender identity corresponds with their sex assigned at birth are referred to as cisgender (CG), while those for whom this is not the case are included in the broader group of transgender and TGD individuals. It is particularly important to recognize transgender and TGD individuals, who defy the traditional division into “woman” and “man.” TGD people may identify as “in between” or “outside” the binary categories, combine elements of different identities, or experience their identity as fluid over time. Research on this group is still relatively limited, even though transgender and TGD individuals are among those most vulnerable to violence, rejection, and a lack of understanding from family, peers, and institutions.

Contemporary concepts emphasize that transgender identity is not a mental disorder, but a developmental variation with complex biological and psychosocial underpinnings, and that the primary source of suffering is primarily the reactions of the environment: stigmatization, discrimination, violence, rejection, systemic barriers, and not the gender identity itself [3,4]. In clinical practice, support for transgender individuals seeking medical assistance focuses on aligning the body with gender identity (e.g., hormone therapy, surgical procedures), while also taking into account the context of trauma and minority stress. People experiencing gender identity incongruence are increasingly seeking psychological and psychiatric help not directly related to their experienced gender dysphoria or identity issues, but rather due to symptoms of depression, anxiety, and signs indicating past traumatic experiences [5].

### 1.2. Childhood Trauma and Adverse Childhood Experiences

Childhood trauma is one of the key concepts in contemporary developmental and clinical psychology. Operationally, and in line with commonly used research tools (e.g., the Childhood Trauma Questionnaire—CTQ), this trauma encompasses five basic forms of maltreatment before the age of 18: emotional, physical, and sexual abuse, as well as emotional and physical neglect. In the literature, these experiences are often included within the more broadly defined category of Adverse Childhood Experiences (ACE), which comprise three main categories: abuse (emotional, physical, sexual), neglect (emotional, physical), and family dysfunction (parental mental illness, addictions, domestic violence, family breakdown, incarceration of a caregiver) [6]. From an interdisciplinary perspective, the concept of childhood trauma also extends to other forms of severe developmental burdens: chronic family conflict, emotional deprivation, boundary violations, emotional manipulation, or a chronic sense of threat in caregiving relationships [7]. Currently, attention is drawn to the distinction between simple trauma (a single, sudden event of high intensity) and complex trauma, which is associated with prolonged violence, neglect, and/or emotional deprivation within the family system [8]. It is complex trauma, especially when rooted in the relationship with caregivers, that appears to be particularly destructive to a child’s development, disrupting the fundamental sense of safety, trust, and coherence of the self.

Epidemiologically, exposure to trauma in childhood is widespread: it is estimated that 30–70% of children experience at least one major traumatic event, with a significant proportion exposed to multiple forms of ACE [6,8]. Within the population of lesbian, gay, bisexual, transgender, queer/questioning, and other identities (LGBTQ+), which encompasses diverse sexual orientations and gender identities (sexual and gender minorities [SGM]), these rates are notably higher. Research showed that 86% of LGBTQ+ youth reported at least one ACE, and 43% reported four or more, with the highest rates observed among transgender and gender nonconforming youth (average ACE-scores of 3.83 and 3.69, respectively) [6]. Similarly, Feil et al. [9] demonstrated that 80% of adults with TGD had experienced at least one ACE, and 29% had experienced multiple traumas, compared to 34% and 6%, respectively, in the cisgender group.

Available neuroimaging data show that different types of trauma cause partially distinct, long-lasting effects on brain function: neglect more often disrupts regions responsible for cognitive functions and emotional control, while violence affects areas responsible for processing emotional stimuli and defense mechanisms [10,11]. It differentiates the pathways leading to various profiles of psychopathology in adulthood. From a clinical perspective, it is emphasized that different forms of trauma have both distinct and overlapping effects: emotional abuse and neglect are most strongly associated with internalizing disorders (especially depression and anxiety). In contrast, physical abuse and neglect more often lead to somatic problems and psychotic symptoms. Experiences of complex, multitype trauma further increase the risk, acting cumulatively and interactively [12,13].

### 1.3. Childhood Trauma and Depression in Transgender and TGD Populations

Trauma experienced in childhood affects mental health in a complex and long-lasting way, through neurobiological and epigenetic changes, disturbances in social functioning and attachment, as well as persistent deficits related to emotional regulation. These effects are modulated by both genetic factors and access to social support [14]. Quantitative and qualitative studies clearly confirm that trauma experienced in childhood significantly increases the risk of depression during adolescence and adulthood [15,16]. Meta-analyses indicate that this risk increases with the number and severity of ACEs, with robust associations related to emotional and sexual abuse [13,15]. The mechanisms linking trauma with depression include both direct effects (neurobiological changes, disturbances in emotion regulation) and indirect effects through reduced psychological resilience, deficits in mentalization, lack of social support, or maladaptive coping strategies [16,17]. Zhang et al. [18] found that smoking, insomnia, and elevated body mass index (BMI) mediate the relationship between physical abuse/physical neglect in childhood and depression in adulthood. This relationship suggests that physical trauma translates into depression through health habits and patterns that promote biological dysregulation.

TGD individuals are particularly vulnerable to childhood trauma. Research on ACE clearly indicates that transgender, nonbinary, and gender nonconforming youth experience significantly more adverse childhood experiences than their cisgender peers, including emotional and physical abuse, neglect, and family dysfunction [6,19]. Feil et al. [9] showed that TGD individuals are twice as likely as cisgender individuals to report at least one ACE (80% vs. 34%), and almost five times as likely to report multiple traumatic and adverse experiences (29% vs. 6%), with the most commonly reported forms being emotional abuse by parents and peer violence (54.3%). The systematic review conducted by Di Fini et al. [19] corroborates that TGD individuals not only encounter ACE with greater frequency but also demonstrate a higher prevalence of depression, anxiety, post-traumatic stress disorder (PTSD), self-harm, and suicidal behaviors.

According to the minority stress model (MSM), TGD individuals experience both distal stressors (discrimination, violence, microaggressions, structural barriers) and proximal stressors (internalized transphobia, expectation of rejection, concealing one’s identity) [20,21,22,23,24,25]. A meta-analysis by Pellicane and Ciesla [26] showed that all these forms of minority stress are clearly associated with depression, suicidal thoughts, and suicide attempts among TGD individuals, with powerful effects for expectation of rejection and internalized transphobia. At the same time, childhood trauma and minority stress rarely occur separately [27,28,29,30,31,32,33]. Research shows that transgender and TGD individuals who have experienced multiple early traumatic events are five times more likely to develop depression, suicidal behaviors, and PTSD symptoms than those without such experiences [9]. McMahon [34] demonstrated that victimization in adulthood partially mediates the relationship between childhood trauma (family dysfunction, emotional abuse, bullying) and psychological distress among individuals from psychosexual and identity minorities, with a lack of social support and identification as transgender and gender-diverse individuals further increasing this risk.

Despite the high burden of trauma and depression, not all transgender and TGD individuals develop mood disorders. Research indicates the significant role of protective factors such as family support, support from friends, connections with the transgender and nonbinary community, self-compassion, and flexible coping strategies [35,36,37]. It has been shown that family support significantly reduces the severity of depression and suicidal thoughts, while a lack of acceptance from loved ones greatly increases the risk of mental health problems and disrupts the process of identity formation [35,36]. In young TGD individuals, a high level of self-compassion and family support is associated with lower levels of depressive symptoms, especially in the youngest age groups [38]. Systematic reviews highlight that the healthcare system itself often becomes a source of secondary traumatization through a lack of competence, prejudice, or ignoring identity, which amplifies the effects of previous trauma and discourages transgender and TGD individuals from seeking help [39,40]. It is advisable to develop trauma-informed care that concurrently affirms gender identity, as this approach may reduce the risk of depression by mitigating the effects of chronic minority stress and trauma [39,40].

Depression is one of the most common mental health disorders among transgender and gender-diverse (TGD) individuals [27,32,33,41]. Studies indicate that the prevalence of depressive symptoms in these groups is significantly higher than that observed among cisgender people [35,42]. Hajek et al. [42] reported that depression symptoms were present in 33.3% of the transgender participants, with the highest severity of symptoms observed among younger individuals, the unemployed, those with poorer self-rated health, and those with coexisting chronic illnesses. According to the minority stress model, chronic exposure to stigmatization, violence, and lack of support (especially from family) constitutes a key mechanism driving the risk of depression in this population [42].

Experiencing traumatic stress in childhood affects neurobiological, behavioral, and psychosocial development throughout a person’s life [27,43]. However, TGD youth experience higher levels of abuse and traumatic stress than their cisgender counterparts [28,29,30,43,44,45]. In particular, TGD individuals experience a significantly greater number of adverse experiences in childhood than CG individuals, in terms of emotional and peer violence as well as family rejection [6,9,19]. These stressors include specific incidents of abuse as well as ongoing, socially ingrained forms of stigma, discrimination, and marginalization, in various environments, such as school, home, and the community [43,46].

Childhood trauma, especially emotional and sexual abuse, is a strong, transdiagnostic risk factor for depression in adulthood, acting through complex neurobiological, psychosocial, and behavioral pathways [11,14,15,16]. In transgender and TGD individuals (nonbinary and gender nonconforming populations), a high prevalence of ACEs co-occurs with a very high burden of depression, anxiety, and PTSD, with minority stress and lack of family support playing key roles [5,26,42].

### 1.4. The Aim of This Study

Although previous research has consistently demonstrated elevated rates of ACEs and depression among TGD populations, most studies remain largely descriptive, focusing on prevalence estimates or bivariate associations. Considerably fewer investigations have examined specific subtypes of childhood trauma within an integrated empirical model that directly compares TGD and CG individuals while exploring potential psychological pathways linking gender identity to depressive symptoms.

Childhood trauma, particularly emotional and sexual abuse, has been identified as a robust transdiagnostic risk factor for depression in adulthood. At the same time, TGD individuals are exposed to cumulative stressors beginning early in life, including family rejection, peer victimization, and gender-based stigma. Despite this overlap, little empirical work has examined whether distinct forms of childhood maltreatment statistically mediate the association between TGD identity and depressive symptom severity.

The present study seeks to address this gap by comparing cisgender and TGD adults in terms of childhood trauma and depressive symptoms and by testing mediation models examining whether overall childhood trauma and its specific subtypes account for group differences in depression. Drawing on Minority Stress Theory and the Temporal Intersectional Minority Stress (TIMS) framework [25,47], we conceptualize early trauma as a potential developmental pathway through which minority-related stress exposure may become biologically and psychologically embedded. We propose that TGD individuals may initiate a minority stress process through both distal and proximal stressors, which are linked to increased abuse and neglect from family members and peers. These traumatic experiences, accumulated during childhood, subsequently impair the mental health of minority populations. Consequently, childhood traumas mediate the relationship between gender identity and depression. Based on previous findings and the TIMS model [25,47], we hypothesize that:

**H1.**  *TGD individuals will report higher levels of adverse childhood experiences and depressive symptoms than the CG sample.*

**H2.**  *All subtypes of childhood trauma positively correlate with depression symptoms in the total sample.*

**Figure 1:**
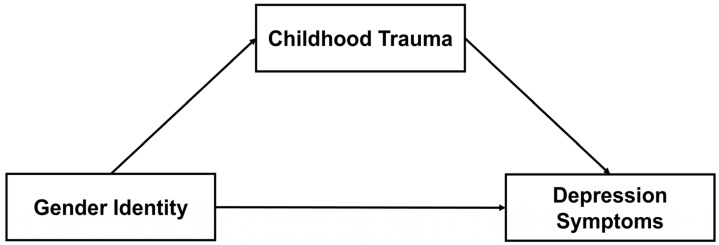
Hypothesized Mediation Model 1 for the indirect effect of gender identity (Cisgender vs. Transgender and Gender-Diverse) on depression symptoms via childhood trauma experiences.

**H3.**  *Overall, childhood trauma mediates the relationship between gender identity (CG vs. TGD) and depression symptoms (Model 1, Figure 1).*

**Figure 2:**
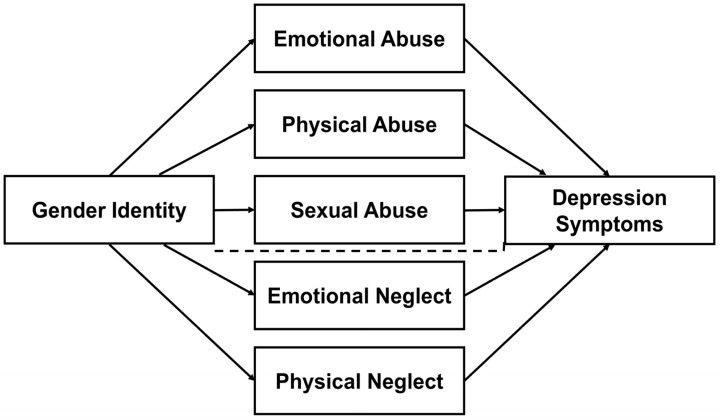
Hypothesized Mediation Model 2 for the indirect effect of gender identity (Cisgender vs. Transgender and Gender-Diverse) on the depression symptoms via particular dimensions of childhood trauma: emotional abuse, physical abuse, and sexual abuse, emotional neglect, and physical neglect. The dashed line represents a non-significant association between Gender Identity and Depression Symptoms, whereas the solid lines indicate significant paths (*p* < 0.05).

**H4.**  *The parallel mediation Model 2 (Figure 2) will examine whether specific trauma subtypes (emotional, physical, and sexual abuse; emotional and physical neglect) uniquely contribute to the association between gender identity and depression.*

While H1 and H2 are confirmatory and consistent with existing literature, the primary contribution of this study lies in the mediation analyses (H3–H4), which aim to clarify trauma-specific pathways linking gender identity and depression. Thus formulated assumptions provide the framework for further empirical analyses and discussion about the specific role of particular subtypes of trauma in the relationship between gender identity and depression.

## 2. Materials and Methods

### 2.1. Study Design and Procedure

The cross-sectional observational research was conducted between 20 May and 1 December 2025, using an online Google Form survey. The sample size was determined using a priori power analysis with G*Power ver. 3.1 software [48]. We aimed for a power of 0.95 with an alpha level of 0.05, testing for a medium effect size. The analysis indicated a minimum required sample size of *N* = 176 for an independent-sample Student’s *t*-test (*n* = 88 per group) and *N* = 149 participants to detect an increase in *R*^2^ with six total predictors in a multiple linear regression to test mediation in Model 2.

The only inclusion criterion was a minimum age of 18 years. Participants were recruited both remotely and in person. The survey was shared on the researchers’ social media with a request for friends to forward it to others. In addition, a link to the survey was sent via email, SMS, and WhatsApp groups (mainly among people working in psychology and psychotherapy) to all contacts, with the same request. In direct contact, the survey was made available via QR code during Equality Marches in Opole, Katowice, Wrocław, Warsaw, and Bielsko-Biała. Individuals, mainly from the LGBT+ community, received free gadgets as a thank-you for participating in the research and for spreading it to others. During mountain rallies in Lower Silesia, participants received printed QR codes in their starter packs.

Additionally, QR codes were distributed throughout the University of Opole in places visible to students, including during classes and training sessions conducted by the researcher. To prevent missing data, all questions were made mandatory by enabling the “Required” option in Google Forms. Initially, 268 people volunteered for the study, but three were under 18, 10 refused to participate, and six dropped out during completion. The final sample included 249 people, which is more than adequate to achieve the expected power of the statistical tests.

### 2.2. Participant Characteristics

The age of study participants ranged from 18 to 72 years (*M* = 30.85, *SD* = 12.72). The majority of participants were women, in a relationship, with a secondary education level, living in a city with more than 100,000 inhabitants (Table 1). For further statistical analyses, we combined cisgender individuals in a CG sample (*n* = 144) and transgender and gender-diverse individuals in the TGD sample (*n* = 105). Considering the cut-off for moderate-to severe experiences, around half of the participants reported current depression symptoms, as well as emotional neglect and emotional abuse during childhood (Table 1). Physical neglect was reported by 43% of the sample, and approximately one-quarter of participants reported experiencing sexual abuse and physical abuse during childhood (Table 1).

### 2.3. Materials

#### 2.3.1. Childhood Trauma

Childhood trauma was measured using the 28-item Childhood Trauma Questionnaire–Short Form (CTQ-SF), developed by Bernstein et al. [49,50,51]. The CTQ-SF is a retrospective screening tool, widely used in a clinical and research setting to understand trauma’s impact on mental health in adolescents and adults. The CTQ-SF is a self-report tool assessing a person’s past experiences with five types of childhood maltreatment: emotional abuse (EA), physical abuse (PA), sexual abuse (SA), emotional neglect (EN), and physical neglect (PN). In addition, the CTQ-SF includes a three-item denial/minimization scale (DE) to assess underreporting. A 5-point response scale (from 1 = *Never true* to 5 = *Very often true*) is used to rate how frequently the given experiences were present during childhood (e.g., “I did not have enough to eat”). The total score ranged between 5 and 25, with higher levels indicating more frequent traumatic experiences during childhood. Moderate to severe cut-off scores are: 13 for Emotional abuse, 10 for Physical abuse, 8 for Sexual abuse, 15 for Emotional neglect, and 10 for Physical neglect [49]. The CTQ-SF demonstrated good to excellent reliability and validity, with Cronbach’s α ranging from 0.61 to 0.95 for particular scales in various research samples [50]. In the present study, Cronbach’s α was 0.89 (95% CI Bc [0.86, 0.91]), 0.82 (95% CI Bc [0.77, 0.86]), 0.92 (95% CI Bc [0.89, 0.94]), 0.93 (95% CI Bc [0.91, 0.95]), 0.65 (95% CI Bc [0.58, 0.71]), 0.60 (95% CI Bc [0.42, 0.73]), and 0.94 (95% CI Bc [0.93, 0.95]), for EA, PA, SA, EN, PN, DE, and the total score of CTQ-SF, respectively. Reliability assessed using McDonald’s ω was respectively 0.89 (95% CI Bc [0.86, 0.91]), 0.84 (95% CI Bc [0.79, 0.87]), 0.93 (95% CI Bc [0.90, 0.95]), 0.93 (95% CI Bc [0.92, 0.95]), 0.68 (95% CI Bc [0.61, 0.74]), 0.64 (95% CI Bc [0.52, 0.98]), and 0.95 (95% CI Bc [0.94, 0.96]). Confirmatory factor analysis (CFA) showed that the five-factor CTQ-SF model fit the data well in the current sample (Supplementary Materials).

#### 2.3.2. Depression Symptoms

The 9-item Patient Health Questionnaire-9 (PHQ-9) is a diagnostic instrument for identifying specific depressive symptoms [52,53]. Participants were instructed to indicate the frequency of each symptom over the preceding 2 weeks, using a 4-point scale from 0 (not at all) to 3 (nearly every day). The total score can range from 0 to 27, with higher scores indicating more severe depressive symptoms. A score of 10 is recognized as the threshold for moderate-to-severe depression. Cronbach’s α in a previous study ranged from 0.86 to 0.89 across various research samples [53], while in this study it is 0.89 (95% CI Bc [0.87, 0.91]), and McDonald’s ω is 0.90 (95% CI Bc [0.87, 0.92]). Confirmatory factor analysis showed that the single-factor model of the PHQ-9 fit the data well in the current sample (Supplementary Materials).

#### 2.3.3. Demographic Survey

The demographic survey included several questions about Age (years old), Gender (categories: Woman, Man, Transgender, Gender-diverse individuals as Other), Place of residence (Village, City with less than 50,000 inhabitants, City with 50,000–100,000 inhabitants, City with more than 100,000 inhabitants), Education (Elementary, Vocational, Secondary, Bachelor’s Degree, Master’s Degree), Relationship status (Single, In a relationship).

### 2.4. Statistical Analysis

A preliminary descriptive analysis was conducted to evaluate the parametric characteristics of depression symptoms and childhood trauma dimensions. Given the relatively large sample size (*N* > 200) and skewness and kurtosis values within the range of −1 to +1, parametric tests were deemed suitable for subsequent analyses, as the data distribution closely approximated normality [54]. To examine gender differences in depression and childhood trauma between the CG vs. TGD samples, the independent samples Student’s *t*-test was employed. Pearson’s correlation was applied to investigate the relationships between depression and childhood trauma (*N* = 249). Lastly, the generalized linear model (GLM) was used for mediation analysis to explore the effect of gender (CG was coded as 0, and TGD was coded as 1) on depression, with childhood trauma serving as the mediator. This model estimates total, direct, and indirect mediation effects using maximum likelihood regression. Confidence intervals (CI) for the estimates were calculated using the bias-corrected bootstrap method with 1000 sample replications. All statistical analyses were conducted using JAMOVI ver. 2.6.44 for Windows.

## 3. Results

### 3.1. Gender Differences in Childhood Trauma and Depression

The independent samples Student’s *t*-test was performed to assess gender differences in particular dimensions of childhood trauma and depression symptoms (Table 2). TGD participants scored higher than the CG sample in symptoms of depression (*p* < 0.001, Cohen’s *d* = −0.69), the overall score of childhood trauma (*p* < 0.001, Cohen’s *d* = −0.85), and all its clinical dimensions, including emotional abuse (*p* < 0.001, Cohen’s *d* = −1.10), physical abuse (*p* = 0.006, Cohen’s *d* = −0.36), sexual abuse (*p* = 0.008, Cohen’s *d* = −0.35), emotional neglect (*p* < 0.001, Cohen’s *d* = −0.88), and physical neglect (*p* < 0.001, Cohen’s *d* = −0.47). In contrast, the TGD sample showed significantly lower levels of denial than CG participants (*p* < 0.001, Cohen’s *d* = 0.55).

As a sensitivity analysis, one-way ANOVA was conducted to examine genders across four categories: Transgender (T), Gender Diverse (GD), Cis Women (CW), and Cis Men (CM) (Supplementary Materials). Significant intergroup differences were found in all scales and subscales of childhood trauma and depression. Transgender and Gender Diverse samples scored higher than Cis Women and Cis Men in depression symptoms, as well as emotional abuse, emotional neglect, and overall childhood trauma. Cis Men scored significantly lower than Transgender and Gender Diverse samples in physical abuse and physical neglect. Also, Cis Men showed significantly lower levels of sexual abuse than the gender diverse group. All details are presented in the Supplementary Materials.

### 3.2. Correlations Between Childhood Trauma and Depression

Pearson’s correlation analysis showed that the total score of childhood trauma is related positively to symptoms of depression at a medium level (Table 3). Among the individual dimensions of childhood trauma, the strongest associations with depressive symptoms were found for emotional abuse, followed by emotional neglect, sexual abuse, physical neglect, and physical abuse (*r* ranged between 0.28 and 0.52). Denial was treated as a covariate to test the validity of the CTQ-SF measure. Denial was negatively related to depression symptoms, emotional abuse, physical abuse, emotional neglect, physical neglect, and the total score of childhood trauma, suggesting that the obtained trauma results are reliable and credible, not distorted by false memories or defense mechanisms, except for sexual abuse (which showed no associations). All dimensions of childhood trauma were interrelated significantly and positively, except denial. The total score of childhood trauma was strongly associated with emotional abuse and emotional neglect (Table 3).

**Figure 3:**
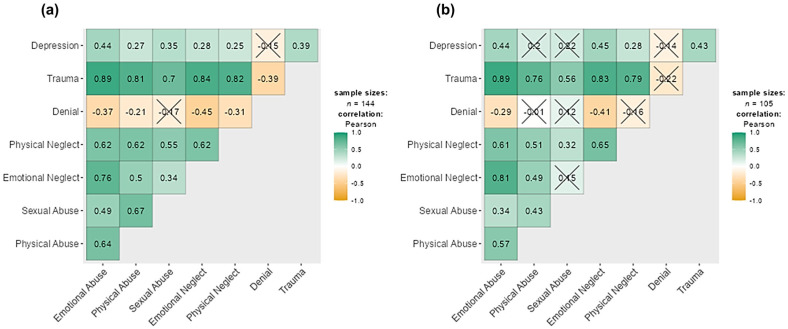
Pearson’s correlations between scales of childhood trauma and depression symptoms across (**a**) cisgender sample, and (**b**) transgender and gender-diverse individuals. Depression = depression symptoms assessed in the Patient Health Questionnaire, Trauma = childhood trauma assessed in the Childhood Trauma Questionnaire. Data marked with a cross indicate non-significant correlations (*p* > 0.05).

Sensitivity analysis was performed to examine the associations between childhood trauma and depression symptoms across the CG and TGD samples (Figure 3). However, the structure and direction of associations are similar in both groups, more insignificant relationships were found in TGD individuals than in CG participants. However, denial was not associated with all variables in the correlation matrix among TGD individuals, except emotional abuse and emotional neglect. This pattern may be interpreted as potentially consistent with defensive response tendencies; however, this interpretation remains hypothetical, and alternative explanations such as differences in reporting style or response tendencies should also be considered.

### 3.3. Mediation Analysis

A GLM mediation analysis was performed to verify Model 1, assuming an indirect effect of gender identity (CG vs. TGD) on depression via childhood trauma (Table 4). The total, direct, and indirect effects were significant, suggesting that the total score of childhood trauma partially mediates the cross-sectional associations between gender and depression symptoms.

In a sensitivity analysis, Model 1a of mediation was repeated with three confounding variables, such as sex assigned at birth (Male = 0, Female = 1), age (ranging between 18 and 72 years), and current education status (Elementary = 0, Vocational = 1, Secondary = 2, Bachelor’s Degree = 3, Master’s Degree = 4). The results showed similar total, direct, and indirect effects as in the previous Model 1 mediation, although of the three confounding variables, only female sex had a significant positive total, direct, and indirect effect on the development of depression symptoms (Supplementary Materials, Model 1a).

The Model 2 was examined using GLM mediation analysis (Table 5). The results suggest that, in this cross-sectional sample, the association between gender identity (CG vs. TGD) and depressive symptoms may be statistically accounted for by reported emotional abuse in childhood. Although sexual abuse showed an insignificant mediating effect (*p* = 0.055), the confidence intervals for bias-corrected bootstrapping, with 1000 sample replications, confirmed that TGD identity contributes to depression symptoms via sexual abuse. In contrast, neither physical abuse, physical neglect, nor emotional neglect was found as a mediator between gender identity and depression symptoms (Table 5).

In the sensitivity analysis, Model 2a of mediation was repeated, including sex assigned at birth, age, and educational status, coded the same as in the previous analysis testing Model 1a. The mediation effect of Emotional Abuse and Sexual Abuse was again confirmed in Model 2a, yielding similar results as in Model 2. Additionally, considering bootstrapping, female sex and younger age were significantly associated with depression via emotional abuse. Female sex also showed an indirect effect on depression symptoms through sexual abuse in the Model 2a mediation (Supplementary Materials, Model 2a).

## 4. Discussion

This study aimed to examine associations between childhood trauma experiences and depression symptoms in a gender diverse sample. As expected, TGD individuals exhibited higher levels of childhood trauma experiences and a greater severity of depressive symptoms compared to CG individuals. Furthermore, based on the TIMS model [25,47], our cross-sectional data suggest that the overall score of childhood trauma partially mediates the association between gender identity (CG vs. TGD) and the severity of depressive symptoms. In particular, the findings indicated that TGD identity has an indirect effect on depression symptoms via emotional and sexual abuse. In contrast, physical abuse and emotional or physical neglect do not predict the severity of depression and cannot serve as mediators.

It is important to note that the results of the CTQ represent reliable and valid outcomes of childhood traumatic experiences. The Denial scale was included in the CTQ-SF as a validation tool for detecting potential underreporting of childhood maltreatment. Because the CTQ relies on retrospective self-report, the authors [49,50] included these three items to identify individuals who may minimize or deny traumatic experiences, often due to psychological defense mechanisms. The primary purpose of including the Denial scale is to detect response bias, particularly underreporting, by identifying respondents who may deny or minimize abuse even if they have experienced it themselves. Furthermore, individuals who have experienced severe trauma may use defense mechanisms such as denial/minimization as a coping mechanism or may be reluctant to disclose abuse due to fear, anxiety, or emotional detachment. The Denial scale also helps detect the tendency to portray parents or caregivers in a falsely positive light (“I had a perfect childhood”), a common psychological reaction to early trauma. High scores on the MD scale suggest a high risk that low levels of abuse/neglect are reported, prompting investigators to seek additional information. Conversely, low scores of denial and a negative correlation between the denial and other CTQ-SF scales suggest that reporting childhood trauma is not subject to measurement error related to the use of denial as a defense mechanism. This study found that denial was negatively associated with all scales of childhood trauma, except sexual abuse, considering the total sample. However, differences were presented between CG and TGD participants. Among TGD individuals, only emotional abuse and emotional neglect were negatively associated with denial, whereas in the CG sample, only the Sexual Abuse scale was unrelated to denial. This pattern may be interpreted as potentially consistent with differences in response tendencies; however, interpretations invoking defensive mechanisms should be considered hypothetical, and alternative explanations related to reporting style cannot be ruled out. In both groups, CG and TGD, Sexual Abuse was unrelated to other traumatic experiences, which may reflect distinct reporting patterns or other unmeasured factors rather than specific psychological mechanisms.

### 4.1. Childhood Trauma Experiences and Depression Across Cisgender, Transgender, and Gender-Diverse Individuals

The study found that people with TGD identity scored significantly higher than the sample of CG individuals in symptoms of depression (medium effect size) and childhood trauma (large effect size for total score). Furthermore, large effect sizes were found for gender differences in emotional abuse and emotional neglect, whereas small effect sizes were observed for sexual abuse, emotional neglect, and physical neglect. The results are consistent with previous literature, which showed that TGD individuals experience more severe depression symptoms and ACE than those with CG identity [6,9,19]. Numerous studies indicate that trauma specific to identity minorities, such as being compelled to adhere to the gender assigned at birth, rejection by loved ones, experiencing transphobia, and peer violence, often co-occur with “classic” ACEs, thereby creating a scenario of dual vulnerability [5,9,39].

Modern models of childhood trauma emphasize its multimodal, transdiagnostic nature. The same early childhood experiences increase the risk of a whole range of mental and somatic disorders, rather than a single, specific diagnosis [14]. These mechanisms encompass temporally associated alterations in neurobiological, epigenetic, psychosocial, and behavioral domains [27,28,43]. The dysregulation of the hypothalamic–pituitary–adrenal (HPA) axis and stress responses, along with an enhanced inflammatory response, contribute to accelerated biological aging. This neurobiological process includes structural and functional changes in the brain, particularly in regions involved in emotion regulation [10,11,55]. Lasting modifications of gene expression can modulate susceptibility to mood disorders, anxiety, and PTSD. Moreover, violence and neglect lead to partially different patterns of these epigenetic changes. In addition, childhood trauma can result in the formation of an insecure or disorganized attachment style. Consequently, it may also lead to the development of negative self-cognitive schemas. Furthermore, low self-esteem and difficulties in emotion regulation are common outcomes. As a result, there is often increased rumination and self-blame following these experiences [15,17]. Individuals exposed to childhood trauma are at greater risk of psychoactive substance use. They may experience sleep disorders and a lack of physical activity. Social isolation is another common consequence of ACE, which, over time, can lead to physical and mental health problems [13,18]. It should be noted that these neurobiological mechanisms were not directly assessed in the present study and are discussed solely on the basis of previous literature.

Childhood trauma is strongly related to increased minority experiences [27,28,29,30,31,32,33]. The model of minority stress explains how cumulative adverse experiences deteriorate well-being and increase risk of mental disorders in the gender and sexual minority population [20,21,22,23,24,25,26]. Furthermore, experiences of victimization in adulthood mediate the link between childhood trauma and psychological distress in individuals belonging to sexual and gender identity minorities [34].

### 4.2. Childhood Trauma as the Primary Pathway to Depression Symptoms

This study showed that TGD identity was associated with depression symptoms, and this association may be statistically accounted for by childhood trauma, particularly emotional and sexual abuse. It should be noted that the same pattern was also observed after including confounding variables, such as sex assigned at birth, age, and education, in mediation models 1a and 2a. The observed pattern is consistent with a growing body of research showing that emotional and sexual abuse are particularly strongly linked to internalizing disorders, especially depression and anxiety, while physical abuse and physical neglect are more often associated with somatic problems and psychotic symptoms [10,12,13]. Our findings support this distinction. Although all forms of trauma were more common among TGD individuals, only those that directly impact the core sense of self-worth, bodily integrity, and attachment to caregivers, namely emotional and sexual abuse, are associated with higher severity of depressive symptoms. From a developmental perspective, emotional abuse by parents and peers, as well as sexual abuse, undermines fundamental assumptions about oneself (e.g., “I am worthless,” “I don’t deserve love”) and about the world (e.g., “others are threatening,” “I can’t trust anyone”).

Research confirms that it is precisely these experiences that contribute to the development of negative cognitive schemas, low self-esteem, difficulties in emotional regulation, and tendencies toward rumination and self-blame, which are key factors in the development of depression [15,16]. Analyses also indicate that among psychopathological symptoms, depression and anxiety constitute the most “central” nodes in the network of connections between trauma and psychopathology in adults [12], which is consistent with the results of our study. On the other hand, the lack of significant mediating effects for physical violence and neglect may reflect two issues. First, previous research suggests that these forms of trauma may be more strongly associated with other areas of psychopathology (e.g., somatization, conduct disorders, psychotic symptoms), and their connection to depression is more indirect (e.g., through health behaviors such as smoking, sleep disorders, or BMI) [18]. Secondly, their impact on depression may only become apparent in the presence of other mediating factors that were not included in this study (e.g., social support, attachment style, and current minority stress).

### 4.3. Results in Light of the Minority Stress Model and Research on TGD Individuals

The results obtained fall within a broad range of studies indicating that TGD individuals experience significantly more adverse and traumatic events in childhood than CG persons, and particularly often report emotional abuse from family members as well as peer violence [6,9,19]. In our study, this pattern is evident in the form of significant differences between CG and TGD individuals in terms of emotional and sexual violence, while these forms of trauma are also linked to depression. According to the minority stress model [25,56], people with TGD identity experience both distal stressors, such as discrimination, violence, rejection, and microaggressions, as well as proximal stressors, including internalized transphobia, expectation of rejection, and the need to hide one’s identity. Some of these burdens have roots as early as childhood and may be related to emotional and sexual abuse. Experiencing gender nonconformity may lead to increased instances of emotional and sexual abuse, including punishment for a child’s gender identity that does not align with parental expectations, and coercion into behaviors deemed “consistent” with the gender assigned at birth. In light of studies on ACE in TGD individuals, it can be assumed that in many cases, this violence is directly related to the environment’s reaction to the developing gender identity or expression [5,9,19].

On the other hand, our results do not support the narrative that being TGD individuals themselves is a factor generating depression. Instead, the study indicates that specific patterns of traumatization, especially relational trauma during childhood, can mediate the associations between gender identity and depression. The study’s findings are consistent with systematic reviews that have shown that higher rates of depression, anxiety, and suicidal behavior occur among TGD individuals as a result of experiencing violence, rejection, and transphobia, rather than gender dysphoria itself [5,19,26]. The study’s results can also be considered in the context of neurobiological data. Studies provide evidence of the long-term impact of childhood trauma on the brain’s functional dynamics, as well as specific changes in neural networks responsible for emotion regulation and the processing of threatening stimuli [11,55]. Significantly, different subtypes of trauma (violence vs. neglect) are associated with distinct patterns of functional and structural changes, which may partially explain why, in our study, it was emotional and sexual violence rather than neglect that was linked to depression. Considering that TGD individuals are exposed to a cumulative burden of trauma, both in childhood and adulthood (victimization, violence, discrimination), it can be assumed that their stress regulation system is particularly strained [34,39]. The results of our study, therefore, fit into a broader picture, according to which early childhood emotional and sexual trauma may contribute to neurobiological vulnerability to depression, which is then reinforced by later experiences of minority stress.

### 4.4. Clinical and Practical Implications

The findings underscore the importance of routinely assessing childhood trauma (especially emotional and sexual abuse) in mental health care for TGD individuals presenting with depressive symptoms. Trauma-informed and gender-affirming clinical approaches are essential, as depressive symptomatology may reflect long-standing relational adversity rather than solely current gender-related stress.

Preventive efforts should prioritize family-based psychoeducation, early support for gender-diverse youth, and interventions promoting caregiver acceptance. Strengthening protective factors such as family support, peer connection, and community belonging may substantially mitigate long-term mental health risks.

At the policy level, these results highlight the need for trauma-informed, affirming health care and educational environments. Structural interventions aimed at reducing discrimination, improving provider competence, and expanding access to supportive services may help interrupt developmental trajectories linking early adversity to adult depressive symptoms.

### 4.5. Limitations of the Study and Directions of Future Studies

Several limitations of this study should be highlighted. First, purposive and convenience sampling were used (participants in online surveys recruited, among others, via social media, LGBT+ community spaces, Pride events, and university contexts), which limits the ability to generalize the results to the broader TGD and CG populations. The sample structure (overrepresentation of more engaged individuals with internet access and ties to queer spaces) may affect both the frequency of trauma and the prevalence of depression.

Second, the study is cross-sectional, which prevents a conclusion about the direction of relationships when using the applied mediation models. Although the hypothesis that childhood trauma precedes the development of depression is well established both theoretically and empirically, more complex pathways cannot be ruled out, for example, the influence of current psychological state on how childhood experiences are recalled and reported.

Third, both trauma and depression were assessed using self-report questionnaires, which makes the results susceptible to memory biases, social desirability bias, response biases (mood, interpretation), and demand characteristics (guessing the study aims). The CTQ-SF and PHQ-9 are widely used, well-studied instruments. However, their validity in TGD populations has not yet been systematically verified, and the lack of adaptations addressing the specifics of minority stress may affect the results. Future studies should consider conducting experimental or qualitative research to address the issues mentioned above.

Fourth, due to the sample size and the nature of the project, a number of potential confounding variables were not taken into account, including sexual orientation, socioeconomic status, current experiences of discrimination, social support, resilience level, and existing mental health disorders. These factors may influence both exposure to trauma and the severity of depression. Our analyses also showed that female sex assigned at birth and age have an indirect effect on depression symptoms, similar to TGD gender identity. Future longitudinal research should examine simultaneously the interactive effects of female sex assigned at birth, current gender identity, and age on depression and individual ACE dimensions.

Fifth, the cross-sectional nature of the study precludes causal inference. Although childhood trauma temporally precedes adult depression, mediation effects should be interpreted strictly as statistical associations. The observed pattern of mediation for emotional abuse may be inflated due to omitted variables, including current minority stress, social support, attachment style, and prior psychopathology. Future research should prioritize longitudinal designs, include indicators of minority stress and protective factors (e.g., family acceptance), and use trauma-informed qualitative approaches. Such work would allow for more nuanced developmental modeling of how early adversity interacts with ongoing gender-related stress to shape mental health trajectories in TGD populations.

Sixth, in the analyses, TGD individuals were combined into a single group that included both binary and non-binary participants. Although it increased statistical power, it simultaneously limited the ability to capture sensitive differences between particular subgroups (e.g., trans-women vs. trans-men vs. non-binary individuals). The literature suggests that these groups may differ both in the profile of traumatic and adverse experiences in childhood, and in depression indicators, which is worth investigating in future projects.

Given the aforementioned limitations, future research should employ longitudinal studies to facilitate the temporal tracking of the relationships among trauma, minority stress, social support, and depression. It is necessary to include cross-cultural research in larger, more diverse samples, which would enable the analysis of differences among individual TGD subgroups and comparisons with cisgender individuals across countries. Furthermore, a multidimensional assessment of trauma should be incorporated, encompassing both classical traumas and childhood adversities, as well as experiences unique to identity minorities, such as being compelled to conform to gender norms and family rejection due to identity. Additionally, the examination of protective factors, such as family support and TGD individuals’ community support, as moderators and mediators of the impact of trauma on depression is essential. Experimentally manipulating or assessing factors such as minority stress, social support, and attachment style could be instrumental in exploring their causal roles in the progression from childhood trauma to adult depression. Employing experimental paradigms, such as stress induction and social support manipulation, appears particularly valuable for elucidating the mechanisms underlying mental health outcomes in TGD populations. Finally, the development and validation of tools for assessing depression and trauma, tailored to the specificities of TGD individuals, should be prioritized, including adaptations of the PHQ-9 and CTQ with consideration of minority stress.

## 5. Conclusions

The results of this study indicate that TGD individuals experience higher levels of early childhood trauma and depression symptoms than cisgender individuals, confirming the existence of significant disparities in mental health. A key finding is that the relationship between TGD identity and depression is partially explained by emotional and sexual abuse in childhood, whereas physical abuse and neglect, although common among TGD individuals, do not play a significant mediating role in this association. The findings support the thesis that it is specific patterns of relational traumatization, rather than gender identity itself, that constitute the main pathway leading to the higher burden of depression in this population. This research expanded the understanding of the minority stress model by including the thread of childhood trauma as a bridge between TGD identity and the cumulative adverse reaction of the immediate environment (family, school), which may increase the risk of depression. The study emphasizes the need to integrate the perspective of childhood trauma and minority stress in research on the mental health of TGD individuals, as well as the need to implement identity-affirming models of systemic care and social support. Practically speaking, this study points to the importance of screening for emotional and sexual trauma, strengthening protective resources (especially family and social support), and developing interventions focused on emotional regulation and rebuilding self-esteem among TGD individuals with a history of early childhood violence.

## Figures and Tables

**Table 1:** Demographic characteristics of participants (*N* = 249).

Variable	Categories of Variable	*n*	%
Gender	Cisgender woman	75	30.10
	Cisgender man	69	27.70
	Transgender	44	17.70
	Gender-diverse	61	24.50
Relationship status	Single	124	49.80
	In a relationship	125	50.20
Education	Elementary	18	7.20
	Vocational	5	2.00
	Secondary	110	44.20
	Bachelor’s Degree	37	14.90
	Master’s Degree	79	31.70
Place of residence	Village	51	20.50
	City with less than 50,000 inhabitants	25	10.00
	City with 50,000–100,000 inhabitants	45	18.10
	City with more than 100,000 inhabitants	128	51.40
Cut-off for moderate-to-severe experiences	Depression symptoms	144	57.80
	Emotional abuse	115	46.20
	Physical abuse	57	22.90
	Sexual abuse	60	24.10
	Emotional neglect	125	50.20
	Physical neglect	108	43.40
Number of childhood traumas	0	92	36.90
	1	28	11.20
	2	30	12.00
	3	46	18.50
	4	26	10.40
	5	27	10.80

**Table 2:** Student’s *t*-test for differences between cisgender vs. transgender and TGD individuals participants in particular dimensions of childhood trauma and depression symptoms.

Variable	CG (*n* = 144)		TGD (*n* = 105)		*t*(247)	*p*	*d*
	*M*	*SD*	*M*	*SD*			
Emotional Abuse	10.18	4.83	15.67	5.18	−8.58	<0.001	−1.10
Physical Abuse	7.38	3.49	8.71	3.91	−2.77	0.006	−0.36
Sexual Abuse	6.52	3.40	7.86	4.24	−2.67	0.008	−0.35
Emotional Neglect	12.36	5.54	17.09	5.11	−6.87	<0.001	−0.88
Physical Neglect	8.70	3.71	10.44	3.66	−3.67	<0.001	−0.47
Denial	0.44	0.80	0.11	0.37	4.49	<0.001	0.55
Childhood Trauma	45.14	17.17	59.75	17.09	−6.64	<0.001	−0.85
Depression Symptoms	9.45	6.37	13.97	6.88	−5.35	<0.001	−0.69

Note. CG = cisgender identity, TGD = trans and gender-diverse identity.

**Table 3:** Pearson’s correlations between scales of childhood trauma and depression symptoms.

Variable	1	2	3	4	5	6	7
1. Emotional Abuse	—						
2. Physical Abuse	0.61 ***	—					
3. Sexual Abuse	0.44 ***	0.56 ***	—				
4. Emotional Neglect	0.82 ***	0.52 ***	0.29 ***	—			
5. Physical Neglect	0.63 ***	0.59 ***	0.46 ***	0.65 ***	—		
6. Denial	−0.40 ***	−0.18 **	−0.11	−0.47 ***	−0.30 ***	—	
7. Childhood Trauma	0.90 ***	0.78 ***	0.64 ***	0.86 ***	0.81 ***	−0.39 ***	—
8. Depression Symptoms	0.52 ***	0.28 ***	0.32 ***	0.43 ***	0.32 ***	−0.21 ***	0.48 ***

Note. ** *p* < 0.01, *** *p* < 0.001.

**Table 4:** The GLM Mediation analysis (Model 1) for the indirect effect of gender identity on depression symptoms through childhood trauma.

				95% CI				
Type	Effect	*b*	*SE*	LL	UL	β	*z*	*p*
Indirect	Gender ⇒ Trauma ⇒ Depression	2.29	0.47	1.53	3.29	0.16	4.85	<0.001
Component	Gender ⇒ Trauma	14.61	2.19	10.12	19.02	0.39	6.67	<0.001
	Trauma ⇒ Depression	0.16	0.02	0.12	0.20	0.42	7.05	<0.001
Direct	Gender ⇒ Depression	2.23	0.84	0.68	3.85	0.16	2.67	0.010
Total	Gender ⇒ Depression	4.52	0.84	2.93	6.18	0.32	5.36	<0.001

Note. CI = confidence intervals computed with the bias-corrected bootstrap method, LL = lower level, UL = upper level.

**Table 5:** The GLM Mediation analysis to examine the indirect effect of gender identity on depression symptoms through particular dimensions of childhood trauma (Model 2).

				95% CI				
Type	Effect	*b*	*SE*	LL	UL	β	*z*	*p*
Indirect	Gender ⇒ EA ⇒ Depression	2.96	0.80	1.60	4.76	0.21	3.70	<0.001
	Gender ⇒ PA ⇒ Depression	−0.30	0.22	−1.02	0.09	−0.02	−1.37	0.169
	Gender ⇒ SA ⇒ Depression	0.44	0.23	0.07	1.12	0.03	1.92	0.055
	Gender ⇒ EN ⇒ Depression	0.46	0.56	−0.61	1.66	0.03	0.81	0.417
	Gender ⇒ PN ⇒ Depression	−0.14	0.25	−0.70	0.40	−0.01	−0.57	0.569
Component	Gender ⇒ EA	5.49	0.64	4.22	6.80	0.48	8.62	<0.001
	EA ⇒ Depression	0.54	0.13	0.29	0.80	0.44	4.09	<0.001
	Gender ⇒ PA	1.33	0.47	0.33	2.31	0.18	2.83	0.005
	PA ⇒ Depression	−0.23	0.14	−0.54	0.10	−0.12	−1.57	0.116
	Gender ⇒ SA	1.34	0.48	0.37	2.33	0.17	2.77	0.006
	SA ⇒ Depression	0.33	0.12	0.01	0.60	0.18	2.67	0.008
	Gender ⇒ EN	4.73	0.69	3.42	5.98	0.40	6.90	<0.001
	EN ⇒ Depression	0.10	0.12	−0.13	0.34	0.08	0.82	0.414
	Gender ⇒ PN	1.74	0.47	0.79	2.62	0.23	3.69	<0.001
	PN ⇒ Depression	−0.08	0.14	−0.36	0.23	−0.05	−0.58	0.565
Direct	Gender ⇒ Depression	1.12	0.86	−0.54	2.90	0.08	1.29	0.196
Total	Gender ⇒ Depression	4.52	0.84	2.77	6.06	0.32	5.36	<0.001

Note. CI = confidence intervals computed with the bias-corrected bootstrap method, LL = lower level, UL = upper level. EA = emotional abuse, PA = physical abuse, SA = sexual abuse, EN = emotional neglect, PN = physical neglect.

## Data Availability

The data presented in this study are available upon request from the corresponding author due to the sensitive information on gender, which is crucial for understanding the statistical analyses and may potentially result in the identification of participants.

## Supplementary Materials

The following supporting information can be downloaded at: https://www.mdpi.com/article/10.3390/healthcare14050558/s1, S1: One-Way ANOVA for Emotional Abuse; S2: One-Way ANOVA for Physical Abuse; S3: One-Way ANOVA for Sexual Abuse; S4: One-Way ANOVA for Emotional Neglect; S5: One-Way ANOVA for Physical Neglect; S6: One-Way ANOVA for Childhood Trauma (total CTQ-SF); S7: One-Way ANOVA for Depression Symptoms; S8: Confirmatory Factor Analysis—Adverse Childhood Experiences (CTQ-SF); S9: Confirmatory Factor Analysis—Depression symptoms (PHQ-9); S10: GLM Mediation Analysis—Model 1a; S11: GLM Mediation Analysis—Model 2a.

## Abbreviations

The following abbreviations are used in this manuscript:

ACE	Adverse Childhood Experiences
BMI	Body Mass Index
CTQ	Childhood Trauma Questionnaire
LGBTQ+	lesbian, gay, bisexual, transgender, queer/questioning, and other identities
MSM	Minority Stress Model
PHQ	Patient Health Questionnaire
SGM	Sexual and Gender Minorities
TIMS	Temporal Intersectional Minority Stress
TGD	Transgender and Gender-Diverse
