# Supplementary material for: How Childhood Maltreatment Contributes to Explaining Depressive Symptoms in Transgender and Gender-Diverse Individuals
Source: Healthcare (Basel). 2026 Feb 24;14(5):558. doi: 10.3390/healthcare14050558 (PMC12984156; doi:10.3390/healthcare14050558)
Supplement: Supplementary file 1 [file healthcare-14-00558-s001.zip › healthcare-4087070-supplementary.pdf]

SUPPLEMENTARY MATERIALS

How childhood maltreatment contributes to explaining depressive symptoms in transgender and gender-diverse individuals

Arkadiusz Parker, and Aleksandra M. Rogowska

CONTENTS

One-Way ANOVA for Emotional Abuse 2
- One-Way ANOVA (Welch's) 2
- Group Descriptives 2

One-Way ANOVA for Physical Abuse 3
- One-Way ANOVA (Welch's) 3
- Group Descriptives 3
- Post Hoc Tests 3

One-Way ANOVA for Sexual Abuse 4
- One-Way ANOVA (Welch's) 4
- Group Descriptives 4
- Post Hoc Tests 4

One-Way ANOVA for Emotional Neglect 5
- One-Way ANOVA (Welch's) 5
- Group Descriptives 5
- Post Hoc Tests 5

One-Way ANOVA for Physical Neglect 6
- One-Way ANOVA (Welch's) 6
- Group Descriptives 6
- Post Hoc Tests 6

One-Way ANOVA for Childhood Trauma (total CTQ-SF) 7
- One-Way ANOVA (Welch's) 7
- Group Descriptives 7
- Post Hoc Tests 7

One-Way ANOVA for Depression Symptoms 8
- One-Way ANOVA (Welch's) 8
- Group Descriptives 8
- Post Hoc Tests 8

Confirmatory Factor Analysis - Adverse Childhood Experiences (CTQ-SF) 9
Factor Loadings 9
Factor Estimates 11
Model Fit 12
Path Diagram 13
Confirmatory Factor Analysis - Depression symptoms (PHQ-9) 14
Factor Loadings 14
Model Fit 14
Fit Measures 15
Path Diagram 15
GLM Mediation Analysis - Model 1a 16
Models Info 16
Path Model 16
Diagram notes 17
Mediation 17
GLM Mediation Analysis - Model 2a 19
Models Info 19
Path Model 21
Diagram notes 21
Mediation 22
Regressions Results 25
Total effects 25
Mediators Models 25
Dependent variable: Emotional Abuse 25
Dependent variable: Physical Abuse 26
Dependent variable: Sexual Abuse 26
Dependent variable: Emotional Neglect 27
Dependent variable: Physical Neglect 27
Full model effects 28

One-Way ANOVA for Emotional Abuse

ANOVA for Emotional Abuse as a dependent variable and Gender Identity (Transgender, Gender-Diverse, Cis Women, Cis Men) as a factor.

One-Way ANOVA (Welch's)

	F	df1	df2	p
**Emotional Abuse**	28.613	3	124.540	<0.001

Group Descriptives

	Gender K	N	Mean	SD	SE
**Emotional Abuse**	**Transgender**	44	15.773	5.220	0.787
	**Cis Men**	69	9.087	4.231	0.509
	**Cis Women**	75	11.187	5.154	0.595
	**Gender Diverse**	61	15.590	5.185	0.664

Post Hoc Tests

Tukey Post-Hoc Test – Emotional Abuse

		Transgender	Cis Men	Cis Women	Gender Diverse
**Transgender**	**p-value**	—	<0.001	<0.001	0.998
**Cis Men**	**p-value**		—	0.055	<0.001
**Cis Women**	**p-value**			—	<0.001
**Gender Diverse**	**p-value**				—

Note. * p < 0.05, ** p < 0.01, *** p < 0.001

One-Way ANOVA for Physical Abuse

ANOVA for Physical Abuse as a dependent variable and Gender Identity (Transgender, Gender-Diverse, Cis Women, Cis Men) as a factor.

One-Way ANOVA (Welch's)

	F	df1	df2	p
**Physical Abuse**	4.114	3	123.212	0.008

Group Descriptives

	Gender K	N	Mean	SD	SE
**Physical Abuse**	**Transgender**	44	8.795	4.015	0.605
	**Cis Men**	69	6.870	2.930	0.353
	**Cis Women**	75	7.840	3.891	0.449
	**Gender Diverse**	61	8.639	3.869	0.495

Post Hoc Tests

Tukey Post-Hoc Test – Physical Abuse

		Transgender	Cis Men	Cis Women	Gender Diverse
**Transgender**	**p-value**	—	0.035	0.518	0.996
**Cis Men**	**p-value**		—	0.388	0.033
**Cis Women**	**p-value**			—	0.587
**Gender Diverse**	**p-value**				—

One-Way ANOVA for Sexual Abuse

ANOVA for Sexual Abuse as a dependent variable and Gender Identity (Transgender, Gender-Diverse, Cis Women, Cis Men) as a factor.

One-Way ANOVA (Welch's)

	F	df1	df2	p
**Sexual Abuse**	5.569	3	120.156	0.001

Group Descriptives

	Gender K	N	Mean	SD	SE
**Sexual Abuse**	**Transgender**	44	7.409	3.902	0.588
	**Cis Men**	69	5.855	2.499	0.301
	**Cis Women**	75	7.133	3.967	0.458
	**Gender Diverse**	61	8.180	4.474	0.573

Post Hoc Tests

Tukey Post-Hoc Test – Sexual Abuse

		Transgender	Cis Men	Cis Women	Gender Diverse
**Transgender**	**p-value**	—	0.141	0.980	0.726
**Cis Men**	**p-value**		—	0.175	0.003
**Cis Women**	**p-value**			—	0.370
**Gender Diverse**	**p-value**				—

One-Way ANOVA for Emotional Neglect

ANOVA for Emotional Neglect as a dependent variable and Gender Identity (Transgender, Gender-Diverse, Cis Women, Cis Men) as a factor.

One-Way ANOVA (Welch's)

	F	df1	df2	p
**Emotional Neglect**	16.240	3	127.015	<0.001

Group Descriptives

	Gender K	N	Mean	SD	SE
**Emotional Neglect**	**Transgender**	44	17.318	5.121	0.772
	**Cis Men**	69	11.638	5.911	0.712
	**Cis Women**	75	13.027	5.120	0.591
	**Gender Diverse**	61	16.918	5.133	0.657

Post Hoc Tests

Tukey Post-Hoc Test – Emotional Neglect

		Transgender	Cis Men	Cis Women	Gender Diverse
**Transgender**	**p-value**	—	<0.001	<0.001	0.982
**Cis Men**	**p-value**		—	0.406	<0.001
**Cis Women**	**p-value**			—	<0.001
**Gender Diverse**	**p-value**				—

One-Way ANOVA for Physical Neglect

ANOVA for Physical Neglect as a dependent variable and Gender Identity (Transgender, Gender-Diverse, Cis Women, Cis Men) as a factor.

One-Way ANOVA (Welch's)

	F	df1	df2	p
**Physical Neglect**	5.838	3	126.025	<0.001

Group Descriptives

	Gender K	N	Mean	SD	SE
**Physical Neglect**	**Transgender**	44	10.455	3.763	0.567
	**Cis Men**	69	8.188	3.336	0.402
	**Cis Women**	75	9.173	3.981	0.460
	**Gender Diverse**	61	10.426	3.617	0.463

Post Hoc Tests

Tukey Post-Hoc Test – Physical Neglect

		Transgender	Cis Men	Cis Women	Gender Diverse
**Transgender**	**p-value**	—	0.009	0.261	1.000
**Cis Men**	**p-value**		—	0.379	0.004
**Cis Women**	**p-value**			—	0.201
**Gender Diverse**	**p-value**				—

One-Way ANOVA for Childhood Trauma (total CTQ-SF)

ANOVA for Childhood Trauma (the total score of CTQ-SF) as a dependent variable and Gender Identity (Transgender, Gender-Diverse, Cis Women, Cis Men) as a factor.

One-Way ANOVA (Welch's)

	F	df1	df2	p
**Trauma**	18.119	3	126.920	<0.001

Group Descriptives

	Gender K	N	Mean	SD	SE
**Trauma**	**Transgender**	44	59.750	16.169	2.438
	**Cis Men**	69	41.638	15.290	1.841
	**Cis Women**	75	48.360	18.253	2.108
	**Gender Diverse**	61	59.754	17.860	2.287

Post Hoc Tests

Tukey Post-Hoc Test – Trauma

		Transgender	Cis Men	Cis Women	Gender Diverse
**Transgender**	**p-value**	—	<0.001	0.003	1.000
**Cis Men**	**p-value**		—	0.086	<0.001
**Cis Women**	**p-value**			—	<0.001
**Gender Diverse**	**p-value**				—

One-Way ANOVA for Depression Symptoms

ANOVA for Depression Symptoms (the total score of PHQ-9) as a dependent variable and Gender Identity (Transgender, Gender-Diverse, Cis Women, Cis Men) as a factor.

One-Way ANOVA (Welch's)

	F	df1	df2	p
**Depression**	12.790	3	124.928	<0.001

Group Descriptives

	Gender K	N	Mean	SD	SE
**Depression**	**Transgender**	44	14.636	6.786	1.023
	**Cis Men**	69	7.971	5.884	0.708
	**Cis Women**	75	10.813	6.528	0.754
	**Gender Diverse**	61	13.492	6.966	0.892

Post Hoc Tests

Tukey Post-Hoc Test – Depression

		Transgender	Cis Men	Cis Women	Gender Diverse
**Transgender**	**p-value**	—	<0.001	0.012	0.811
**Cis Men**	**p-value**		—	0.046	<0.001
**Cis Women**	**p-value**			—	0.083
**Gender Diverse**	**p-value**				—

Confirmatory Factor Analysis - Adverse Childhood Experiences (CTQ-SF)

CFA for Childhood Trauma (CTQ-SF) – five-factor model.

Factor Loadings

				95% Confidence Interval				
Factor	Indicator	Estimate	SE	Lower	Upper	Z	p	Stand. Estimate
**Emotional Abuse**	**CTQ_03**	1.117	0.074	0.972	1.263	15.046	<0.001	0.806
	**CTQ_08**	1.021	0.076	0.873	1.169	13.488	<0.001	0.748
	**CTQ_14**	1.141	0.070	1.003	1.278	16.293	<0.001	0.849
	**CTQ_18**	1.034	0.074	0.889	1.179	13.995	<0.001	0.767
	**CTQ_25**	1.013	0.077	0.862	1.163	13.225	<0.001	0.737
**Physical Abuse**	**CTQ_09**	0.521	0.043	0.436	0.606	12.033	<0.001	0.713
	**CTQ_11**	0.846	0.058	0.731	0.960	14.501	<0.001	0.814
	**CTQ_12**	0.815	0.071	0.677	0.953	11.552	<0.001	0.687
	**CTQ_15**	0.785	0.067	0.652	0.917	11.637	<0.001	0.703
	**CTQ_17**	0.458	0.042	0.376	0.540	10.970	<0.001	0.665
**Sexual Abuse**	**CTQ_20**	0.848	0.051	0.748	0.948	16.663	<0.001	0.854
	**CTQ_21**	0.473	0.048	0.379	0.566	9.906	<0.001	0.584
	**CTQ_23**	0.627	0.040	0.549	0.706	15.675	<0.001	0.821
	**CTQ_24**	0.844	0.044	0.757	0.931	19.017	<0.001	0.923
	**CTQ_27**	0.868	0.043	0.783	0.953	19.969	<0.001	0.949
**Emotional Neglect**	**CTQ_05**	1.065	0.073	0.921	1.209	14.515	<0.001	0.780

Factor Loadings

				95% Confidence Interval				
Factor	Indicator	Estimate	SE	Lower	Upper	Z	p	Stand. Estimate
	**CTQ_07**	1.089	0.068	0.956	1.221	16.122	<0.001	0.836
	**CTQ_13**	1.102	0.063	0.979	1.225	17.554	<0.001	0.881
	**CTQ_19**	1.147	0.068	1.014	1.280	16.899	<0.001	0.861
	**CTQ_28**	1.219	0.065	1.092	1.346	18.772	<0.001	0.917
**Physical Neglect**	**CTQ_01**	0.235	0.089	0.059	0.410	2.621	0.009	0.175
	**CTQ_02**	1.144	0.073	1.000	1.287	15.622	<0.001	0.849
	**CTQ_04**	0.533	0.067	0.402	0.665	7.942	<0.001	0.497
	**CTQ_06**	0.339	0.047	0.247	0.430	7.241	<0.001	0.463
	**CTQ_26**	0.807	0.072	0.665	0.948	11.181	<0.001	0.660

Factor Estimates

Factor Covariances

				95% Confidence Interval				
		Estimate	SE	Lower	Upper	Z	p	Stand. Estimate
**Emotional Abuse**	**Emotional Abuse**	1.000[a]						
	**Physical Abuse**	0.680	0.045	0.592	0.769	15.021	<0.001	0.680
	**Sexual Abuse**	0.457	0.056	0.347	0.567	8.166	<.0001	0.457
	**Emotional Neglect**	0.895	0.020	0.857	0.933	45.847	<0.001	0.895
	**Physical Neglect**	0.843	0.033	0.779	0.907	25.659	<0.001	0.843
**Physical Abuse**	**Physical Abuse**	1.000[a]						
	**Sexual Abuse**	0.585	0.053	0.482	0.689	11.090	<0.001	0.585
	**Emotional Neglect**	0.564	0.052	0.462	0.666	10.844	<0.001	0.564
	**Physical Neglect**	0.686	0.052	0.585	0.787	13.306	<0.001	0.686
**Sexual Abuse**	**Sexual Abuse**	1.000[a]						
	**Emotional Neglect**	0.316	0.061	0.197	0.435	5.206	<0.001	0.316
	**Physical Neglect**	0.508	0.058	0.395	0.622	8.773	<0.001	0.508
**Emotional Neglect**	**Emotional Neglect**	1.000[a]						
	**Physical Neglect**	0.887	0.026	0.836	0.938	33.856	<0.001	0.887
**Physical Neglect**	**Physical Neglect**	1.000[a]						

[a] fixed parameter

Model Fit

Test for Exact Fit

χ^2	df	p
720.344	265	< 0.001

Fit Measures

				RMSEA 90% CI	
CFI	TLI	SRMR	RMSEA	Lower	Upper
0.898	0.884	0.068	0.083	0.076	0.090

Path Diagram

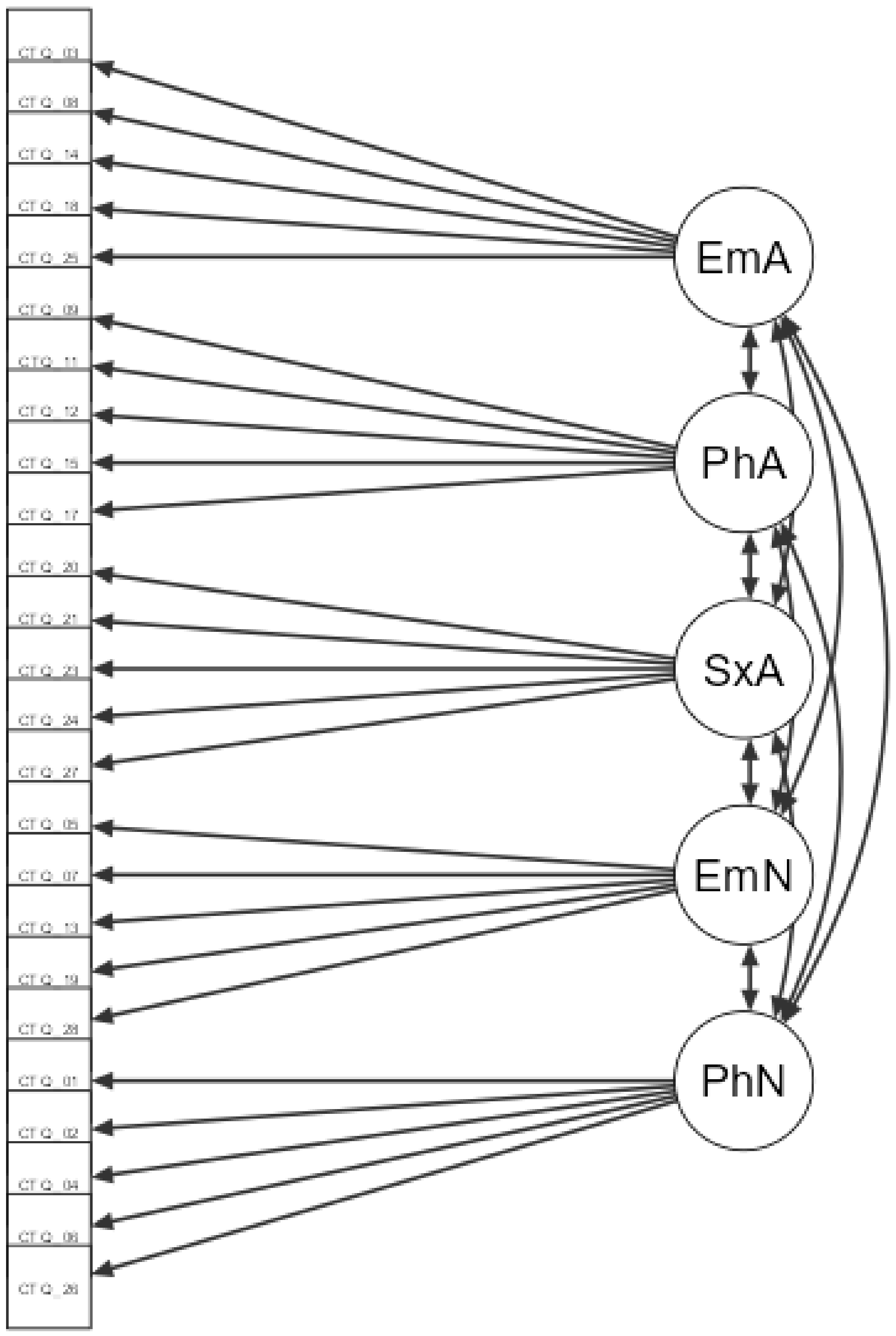

Confirmatory Factor Analysis - Depression symptoms (PHQ-9)

CFA for Depression Symptoms (PHQ-9) – one-factor model.

Factor Loadings

				95% Confidence Interval				
**Factor**	**Indicator**	**Estimate**	**SE**	**Lower**	**Upper**	**Z**	**p**	**Stand. Estimate**
**Factor 1**	**PHQ_01**	0.692	0.057	0.580	0.804	12.089	<0.001	0.696
	**PHQ_02**	0.830	0.055	0.722	0.938	15.038	<0.001	0.812
	**PHQ_03**	0.664	0.068	0.530	0.798	9.704	<0.001	0.585
	**PHQ_04**	0.730	0.058	0.617	0.844	12.602	<0.001	0.717
	**PHQ_05**	0.727	0.064	0.602	0.852	11.404	<0.001	0.667
	**PHQ_06**	0.898	0.061	0.779	1.017	14.806	<0.001	0.802
	**PHQ_07**	0.772	0.064	0.646	0.899	11.973	<0.001	0.690
	**PHQ_08**	0.509	0.053	0.404	0.614	9.518	<0.001	0.576
	**PHQ_09**	0.733	0.058	0.620	0.846	12.680	<0.001	0.721

Model Fit

Test for Exact Fit

χ^2	df	p
72.841	27	<0.001

Fit Measures

				RMSEA 90% CI	
CFI	TLI	SRMR	RMSEA	Lower	Upper
0.955	0.940	0.037	0.083	0.060	0.106

Path Diagram

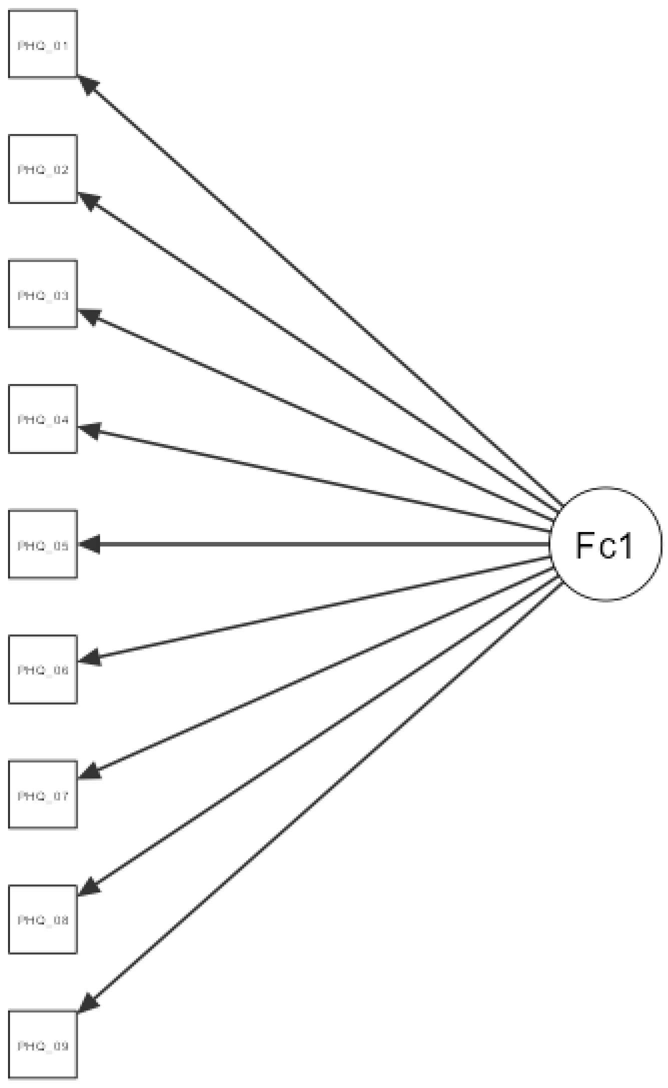

GLM Mediation Analysis - Model 1a

Mediation Model 1a, testing indirect effect of gender identity on depression symptoms via the overall score of childhood trauma, with sex assigned at birth, age, and educational status as confounders.

Models Info

**Mediators Models**		
	**m1**	**Trauma ~ Gender + Sex + Age + Education**
**Full Model**		
	**m2**	**Depression ~ Trauma + Gender + Sex + Age + Education**
**Indirect Effects**		
	**IE 1**	**Gender ⇒ Trauma ⇒ Depression**
	**IE 2**	**Sex ⇒ Trauma ⇒ Depression**
	**IE 3**	**Age ⇒ Trauma ⇒ Depression**
	**IE 4**	**Education ⇒ Trauma ⇒ Depression**
**Sample size**	**N**	**249**

Path Model

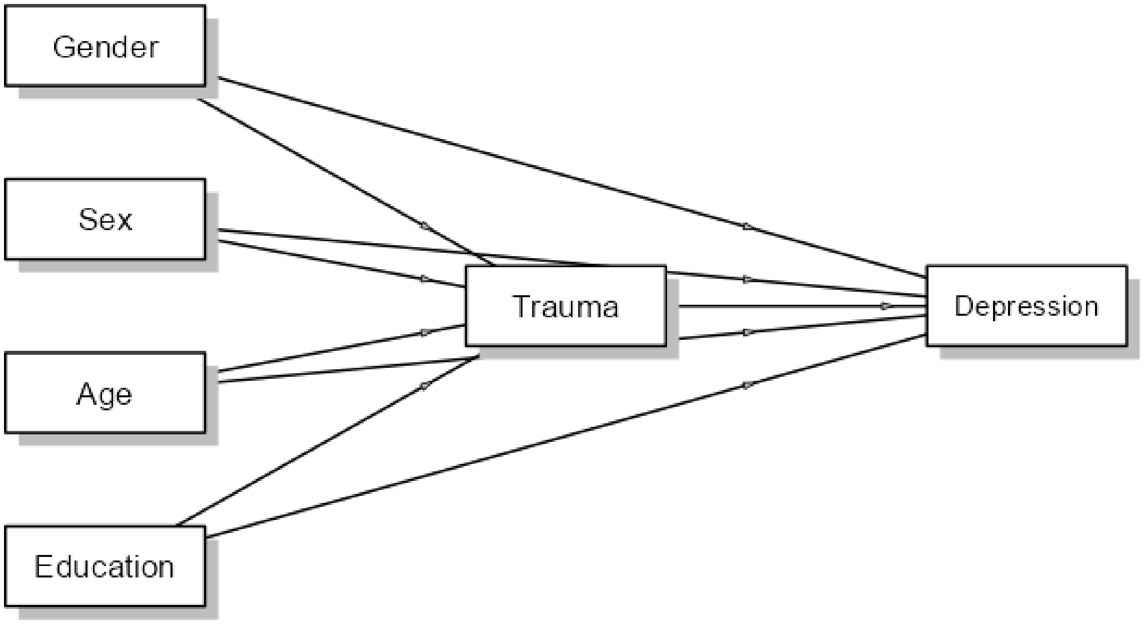

Diagram notes

Categorical independent variables (factors) are shown with only one rectangle, but their effect is estimated using contrast variables

For variable **Gender** the contrasts are: Gender1 = 1 - 0

Covariances among IV are estimated but not shown

Mediation

Indirect and Total Effects

				95% C.I. (a)				
**Type**	**Effect**	**Estimate**	**SE**	**Lower**	**Upper**	**β**	**z**	**p**
**Indirect**	**Gender1 ⇒ Trauma ⇒ Depression**	2.312	0.523	1.479	3.407	0.165	4.419	<0.001
	**Sex ⇒ Trauma ⇒ Depression**	1.018	0.427	0.268	1.977	0.067	2.384	0.017
	**Age ⇒ Trauma ⇒ Depression**	-0.015	0.015	-0.041	0.010	-0.028	-1.046	0.296
	**Education ⇒ Trauma ⇒ Depression**	-0.181	0.156	-0.538	0.086	-0.030	-1.160	0.246
**Component**	**Gender1 ⇒ Trauma**	16.507	2.688	11.240	21.567	0.440	6.142	<0.001
	**Trauma ⇒ Depression**	0.140	0.022	0.100	0.181	0.375	6.363	<0.001
	**Sex ⇒ Trauma**	7.270	2.828	1.682	12.295	0.180	2.571	0.010
	**Age ⇒ Trauma**	-0.111	0.104	-0.275	0.078	-0.076	-1.060	0.289
	**Education ⇒ Trauma**	-1.296	1.098	-3.418	0.698	-0.081	-1.180	0.238
**Direct**	**Gender1 ⇒ Depression**	2.587	1.002	0.842	4.699	0.184	2.583	0.010
	**Sex ⇒ Depression**	2.345	0.995	0.663	4.550	0.155	2.356	0.018

Indirect and Total Effects

Type	Effect	Estimate	SE	95% C.I. (a) Lower	Upper	β	z	p
	**Age ⇒ Depression**	-0.093	0.036	-0.164	-0.029	-0.171	-2.560	0.010
	**Education ⇒ Depression**	-0.353	0.383	-1.088	0.407	-0.059	-0.922	0.356
**Total**	**Gender1 ⇒ Depression**	4.900	1.009	2.891	6.928	0.349	4.858	<0.001
	**Sex ⇒ Depression**	3.363	1.061	1.255	5.356	0.223	3.169	0.002
	**Age ⇒ Depression**	-0.108	0.039	-0.177	-0.043	-0.199	-2.770	0.006
	**Education ⇒ Depression**	-0.534	0.412	-1.246	0.176	-0.089	-1.296	0.195

Note. Confidence intervals computed with method: Bias corrected bootstrap

Note. Betas are completely standardized effect sizes

GLM Mediation Analysis - Model 2a

Parallel Mediation Model 2a, testing indirect effect of gender identity on depression symptoms via the five dimensions of childhood trauma (emotional abuse, physical abuse, sexual abuse, emotional neglect, and physical neglect), with sex assigned at birth, age, and educational status as confounders.

Models Info

**Mediators Models**		
	**m1**	**Emotional Abuse ~ Gender + Sex + Age + Education**
	**m2**	**Physical Abuse ~ Gender + Sex + Age + Education**
	**m3**	**SexdWFsIEFidXNl ~ Gender + Sex + Age + Education**
	**m4**	**Emotional Neglect ~ Gender + Sex + Age + Education**
	**m5**	**Physical Neglect ~ Gender + Sex + Age + Education**
**Full Model**		
	**m6**	**Depression ~ Emotional Abuse + Physical Abuse + SexdWFsIEFidXNl + Emotional Neglect + Physical Neglect + Gender + Sex + Age + Education**
**Indirect Effects**		
	**IE 1**	**Gender ⇒ Emotional Abuse ⇒ Depression**
	**IE 2**	**Gender ⇒ Physical Abuse ⇒ Depression**
	**IE 3**	**Gender ⇒ Sexual Abuse ⇒ Depression**
	**IE 4**	**Gender ⇒ Emotional Neglect ⇒ Depression**
	**IE 5**	**Gender ⇒ Physical Neglect ⇒ Depression**
	**IE 6**	**Sex ⇒ Emotional Abuse ⇒ Depression**
	**IE 7**	**Sex ⇒ Physical Abuse ⇒ Depression**

Models Info

	**IE 8**	**Sex ⇒ Sexual Abuse ⇒ Depression**
	**IE 9**	**Sex ⇒ Emotional Neglect ⇒ Depression**
	**IE 10**	**Sex ⇒ Physical Neglect ⇒ Depression**
	**IE 11**	**Age ⇒ Emotional Abuse ⇒ Depression**
	**IE 12**	**Age ⇒ Physical Abuse ⇒ Depression**
	**IE 13**	**Age ⇒ Sexual Abuse ⇒ Depression**
	**IE 14**	**Age ⇒ Emotional Neglect ⇒ Depression**
	**IE 15**	**Age ⇒ Physical Neglect ⇒ Depression**
	**IE 16**	**Education ⇒ Emotional Abuse ⇒ Depression**
	**IE 17**	**Education ⇒ Physical Abuse ⇒ Depression**
	**IE 18**	**Education ⇒ Sexual Abuse ⇒ Depression**
	**IE 19**	**Education ⇒ Emotional Neglect ⇒ Depression**
	**IE 20**	**Education ⇒ Physical Neglect ⇒ Depression**
**Sample size**	**N**	**249**

Path Model

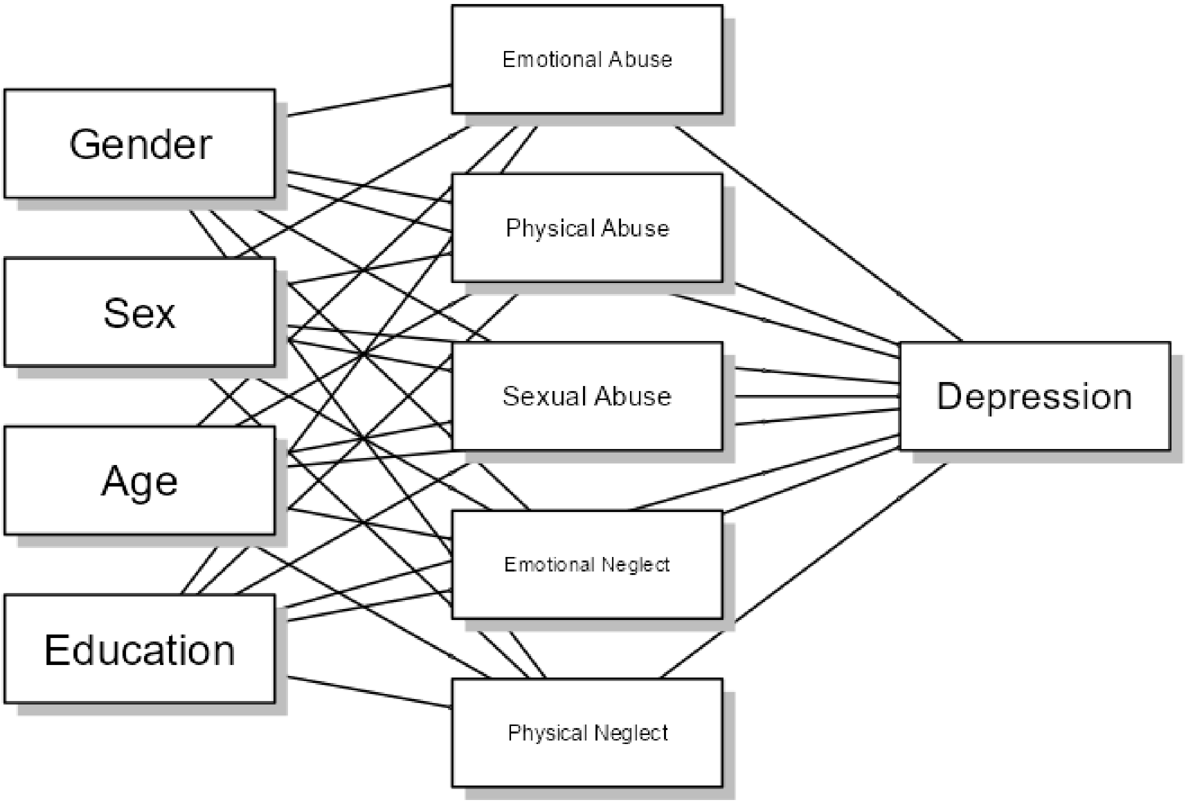

Diagram notes

Categorical independent variables (factors) are shown with only one rectangle, but their effect is estimated using contrast variables

For variable **Gender** the contrasts are: Gender1 = 1 - 0

Covariances among IV are estimated but not shown

Mediation

Indirect and Total Effects

				95% C.I. (a)				
**Type**	**Effect**	**Estimate**	**SE**	**Lower**	**Upper**	**β**	**z**	**p**
**Indirect**	**Gender1 ⇒ Emotional Abuse ⇒ Depression**	2.316	0.847	0.682	4.129	0.165	2.735	0.006
	**Gender1 ⇒ Physical Abuse ⇒ Depression**	-0.240	0.293	-0.992	0.368	-0.017	-0.818	0.413
	**Gender1 ⇒ Sexual Abuse ⇒ Depression**	0.564	0.287	0.120	1.201	0.040	1.963	0.050
	**Gender1 ⇒ Emotional Neglect ⇒ Depression**	0.733	0.600	-0.425	2.294	0.052	1.222	0.222
	**Gender1 ⇒ Physical Neglect ⇒ Depression**	-0.220	0.279	-0.900	0.250	-0.016	-0.789	0.430
	**Sex ⇒ Emotional Abuse ⇒ Depression**	0.988	0.466	0.256	2.182	0.065	2.119	0.034
	**Sex ⇒ Physical Abuse ⇒ Depression**	-0.116	0.157	-0.710	0.107	-0.008	-0.740	0.459
	**Sex ⇒ Sexual Abuse ⇒ Depression**	0.401	0.249	0.050	1.069	0.027	1.609	0.108
	**Sex ⇒ Emotional Neglect ⇒ Depression**	0.219	0.218	-0.070	1.108	0.015	1.003	0.316
	**Sex ⇒ Physical Neglect ⇒ Depression**	-0.121	0.165	-0.712	0.094	-0.008	-0.735	0.462
	**Age ⇒ Emotional Abuse ⇒ Depression**	-0.030	0.016	-0.073	-0.006	-0.055	-1.919	0.055
	**Age ⇒ Physical Abuse ⇒ Depression**	-0.001	0.003	-0.013	0.004	-0.001	-0.187	0.852
	**Age ⇒ Sexual Abuse ⇒ Depression**	-0.001	0.007	-0.016	0.010	-0.003	-0.207	0.836
	**Age ⇒ Emotional Neglect ⇒ Depression**	-0.003	0.005	-0.029	0.004	-0.006	-0.600	0.549
	**Age ⇒ Physical Neglect ⇒ Depression**	0.001	0.003	-0.004	0.018	0.003	0.456	0.648

Indirect and Total Effects

Type	Effect	Estimate	SE	95% C.I. (a) Lower	Upper	β	z	p
	**Education ⇒ Emotional Abuse ⇒ Depression**	-0.176	0.139	-0.553	0.035	-0.030	-1.271	0.204
	**Education ⇒ Physical Abuse ⇒ Depression**	-0.016	0.035	-0.190	0.043	-0.003	-0.466	0.641
	**Education ⇒ Sexual Abuse ⇒ Depression**	-0.061	0.079	-0.289	0.052	-0.010	-0.781	0.435
	**Education ⇒ Emotional Neglect ⇒ Depression**	-0.058	0.069	-0.300	0.043	-0.010	-0.847	0.397
	**Education ⇒ Physical Neglect ⇒ Depression**	0.045	0.062	-0.041	0.270	0.008	0.728	0.466
**Component**	**Gender1 ⇒ Emotional Abuse**	5.772	0.763	4.396	7.178	0.504	7.562	<0.001
	**Emotional Abuse ⇒ Depression**	0.401	0.137	0.112	0.660	0.327	2.933	0.003
	**Gender1 ⇒ Physical Abuse**	1.952	0.584	0.845	2.996	0.259	3.340	<0.001
	**Physical Abuse ⇒ Depression**	-0.123	0.145	-0.439	0.219	-0.066	-0.844	0.399
	**Gender1 ⇒ Sexual Abuse**	1.839	0.598	0.700	2.908	0.238	3.075	0.002
	**Sexual Abuse ⇒ Depression**	0.307	0.120	0.043	0.557	0.169	2.551	0.011
	**Gender1 ⇒ Emotional Neglect**	5.033	0.849	3.376	6.855	0.427	5.931	<0.001
	**Emotional Neglect ⇒ Depression**	0.146	0.117	-0.093	0.408	0.122	1.249	0.212
	**Gender1 ⇒ Physical Neglect**	1.912	0.582	0.840	2.998	0.250	3.287	0.001
	**Physical Neglect ⇒ Depression**	-0.115	0.142	-0.385	0.166	-0.063	-0.813	0.416
	**Sex ⇒ Emotional Abuse**	2.462	0.803	0.981	3.937	0.200	3.066	0.002
	**Sex ⇒ Physical Abuse**	0.947	0.615	-0.182	2.046	0.117	1.540	0.124
	**Sex ⇒ Sexual Abuse**	1.305	0.629	0.158	2.440	0.157	2.074	0.038
	**Sex ⇒ Emotional Neglect**	1.504	0.893	-0.120	3.363	0.118	1.684	0.092

Indirect and Total Effects

Type	Effect	Estimate	SE	95% C.I. (a) Lower	Upper	β	z	p
	**Sex ⇒ Physical Neglect**	1.052	0.612	-0.139	2.239	0.128	1.719	0.086
	**Age ⇒ Emotional Abuse**	-0.075	0.030	-0.126	-0.024	-0.169	-2.538	0.011
	**Age ⇒ Physical Abuse**	0.004	0.023	-0.034	0.046	0.015	0.192	0.848
	**Age ⇒ Sexual Abuse**	-0.005	0.023	-0.041	0.033	-0.016	-0.208	0.835
	**Age ⇒ Emotional Neglect**	-0.023	0.033	-0.086	0.038	-0.049	-0.684	0.494
	**Age ⇒ Physical Neglect**	-0.012	0.023	-0.057	0.039	-0.042	-0.551	0.582
	**Education ⇒ Emotional Abuse**	-0.440	0.312	-0.987	0.226	-0.090	-1.410	0.159
	**Education ⇒ Physical Abuse**	0.133	0.239	-0.315	0.562	0.042	0.559	0.576
	**Education ⇒ Sexual Abuse**	-0.200	0.244	-0.641	0.216	-0.061	-0.820	0.412
	**Education ⇒ Emotional Neglect**	-0.400	0.347	-1.020	0.269	-0.080	-1.152	0.249
	**Education ⇒ Physical Neglect**	-0.389	0.238	-0.900	0.078	-0.120	-1.638	0.101
**Direct**	**Gender1 ⇒ Depression**	1.745	1.025	-0.069	3.634	0.124	1.703	0.089
	**Sex ⇒ Depression**	1.993	0.986	0.106	3.982	0.132	2.021	0.043
	**Age ⇒ Depression**	-0.074	0.037	-0.148	-0.009	-0.137	-2.031	0.042
	**Education ⇒ Depression**	-0.267	0.381	-1.018	0.490	-0.045	-0.700	0.484
**Total**	**Gender1 ⇒ Depression**	4.900	1.009	3.071	6.978	0.349	4.858	<0.001
	**Sex ⇒ Depression**	3.363	1.061	1.354	5.453	0.223	3.169	0.002
	**Age ⇒ Depression**	-0.108	0.039	-0.182	-0.043	-0.199	-2.770	0.006
	**Education ⇒ Depression**	-0.534	0.412	-1.215	0.265	-0.089	-1.296	0.195

Note. Confidence intervals computed with method: Bias corrected bootstrap

Note. Betas are completely standardized effect sizes

Regressions Results

Total effects

ANOVA Table

R-squared	F	df1	df2	p
0.185	13.817	4.000	244.000	<0.001

Total effects predicting: Depression

Names	Effect	Estimate	SE	Lower	Upper	β	df	t	p
Gender1	1 - 0	4.900	1.017	2.897	6.903	0.349	244	4.818	<0.001
Sex	Sex	3.363	1.070	1.255	5.471	0.223	244	3.143	0.002
Age	Age	-0.108	0.039	-0.186	-0.031	-0.199	244	-2.748	0.006
Education	Education	-0.534	0.416	-1.353	0.284	-0.089	244	-1.286	0.200

Mediators Models

Dependent variable: Emotional Abuse

ANOVA

R-squared	F	df1	df2	p
0.295	25.522	4.000	244.000	<0.001

Regression

Names	Effect	Estimate	SE	Lower	Upper	β	df	t	p
Gender1	1 - 0	5.772	0.771	4.253	7.291	0.504	244	7.486	<0.001
Sex	Sex	2.462	0.811	0.864	4.060	0.200	244	3.035	0.003
Age	Age	-0.075	0.030	-0.134	-0.016	-0.169	244	-2.512	0.013
Education	Education	-0.440	0.315	-1.060	0.181	-0.090	244	-1.396	0.164

Dependent variable: Physical Abuse

ANOVA

R-squared	F	df1	df2	p
0.043	2.769	4.000	244.000	0.028

Regression

Names	Effect	Estimate	SE	Lower	Upper	β	df	t	p
Gender1	1 - 0	1.952	0.590	0.789	3.115	0.259	244	3.306	0.001
Sex	Sex	0.947	0.621	-0.277	2.171	0.117	244	1.524	0.129
Age	Age	0.004	0.023	-0.041	0.049	0.015	244	0.190	0.850
Education	Education	0.133	0.241	-0.342	0.609	0.042	244	0.553	0.581

Dependent variable: Sexual Abuse

ANOVA

R-squared	F	df1	df2	p
0.051	3.250	4.000	244.000	0.013

Regression

Names	Effect	Estimate	SE	Lower	Upper	β	df	t	p
Gender1	1 - 0	1.839	0.604	0.649	3.029	0.238	244	3.044	0.003
Sex	Sex	1.305	0.636	0.053	2.558	0.157	244	2.053	0.041
Age	Age	-0.005	0.023	-0.051	0.041	-0.016	244	-0.206	0.837
Education	Education	-0.200	0.247	-0.687	0.286	-0.061	244	-0.812	0.418

Dependent variable: Emotional Neglect

ANOVA

R-squared	F	df1	df2	p
0.180	13.381	4.000	244.000	<0.001

Regression

Names	Effect	Estimate	SE	Lower	Upper	β	df	t	p
Gender1	1 - 0	5.033	0.857	3.344	6.721	0.427	244	5.871	<0.001
Sex	Sex	1.504	0.902	-0.273	3.281	0.118	244	1.667	0.097
Age	Age	-0.023	0.033	-0.088	0.043	-0.049	244	-0.677	0.499
Education	Education	-0.400	0.350	-1.090	0.290	-0.080	244	-1.141	0.255

Dependent variable: Physical Neglect

ANOVA

R-squared	F	df1	df2	p
0.081	5.354	4.000	244.000	<0.001

Regression

Names	Effect	Estimate	SE	Lower	Upper	β	df	t	p
Gender1	1 - 0	1.912	0.587	0.755	3.069	0.250	244	3.254	0.001
Sex	Sex	1.052	0.618	-0.166	2.270	0.128	244	1.702	0.090
Age	Age	-0.012	0.023	-0.057	0.032	-0.042	244	-0.545	0.586
Education	Education	-0.389	0.240	-0.862	0.084	-0.120	244	-1.621	0.106

Full model effects

ANOVA Table

R-squared	F	df1	df2	p
0.326	12.856	9.000	239.000	<0.001

Full model predicting Depression

Names	Effect	Estimate	SE	Lower	Upper	β	df	t	p
Emotional Abuse	Emotional Abuse	0.401	0.140	0.126	0.676	0.327	239	2.874	0.004
Physical Abuse	Physical Abuse	-0.123	0.148	-0.415	0.170	-0.066	239	-0.827	0.409
Sexual Abuse	Sexual Abuse	0.307	0.123	0.065	0.549	0.169	239	2.499	0.013
Emotional Neglect	Emotional Neglect	0.146	0.119	-0.089	0.380	0.122	239	1.224	0.222
Physical Neglect	Physical Neglect	-0.115	0.145	-0.400	0.170	-0.063	239	-0.797	0.426
Gender1	1 - 0	1.745	1.046	-0.315	3.806	0.124	239	1.669	0.097
Sex	Sex	1.993	1.006	0.010	3.975	0.132	239	1.980	0.049
Age	Age	-0.074	0.037	-0.148	-0.001	-0.137	239	-1.990	0.048
Education	Education	-0.267	0.389	-1.032	0.499	-0.045	239	-0.686	0.494
